# Classification of self-assembling protein nanoparticle architectures for applications in vaccine design

**DOI:** 10.1098/rsos.161092

**Published:** 2017-04-26

**Authors:** G. Indelicato, P. Burkhard, R. Twarock

**Affiliations:** 1 Dipartimento di Matematica, Università di Torino, Via Carlo Alberto 10, 10123 Torino, Italy; 2 The Institute of Materials Science, University of Connecticut, 97 North Eagleville Road, Storrs, CT 06269, USA; 3 Department of Molecular and Cell Biology, University of Connecticut, Storrs, CT, 06269-3125, USA; 4 Departments of Mathematics and Biology, University of York, York YO10 5DD, UK; 5 York Centre for Complex Systems Analysis, University of York, York YO10 5GE, UK

**Keywords:** graph theory, symmetry, nanoparticle, fullerene, antigen display

## Abstract

We introduce here a mathematical procedure for the structural classification of a specific class of self-assembling protein nanoparticles (SAPNs) that are used as a platform for repetitive antigen display systems. These SAPNs have distinctive geometries as a consequence of the fact that their peptide building blocks are formed from two linked coiled coils that are designed to assemble into trimeric and pentameric clusters. This allows a mathematical description of particle architectures in terms of bipartite (3,5)-regular graphs. Exploiting the relation with fullerene graphs, we provide a complete atlas of SAPN morphologies. The classification enables a detailed understanding of the spectrum of possible particle geometries that can arise in the self-assembly process. Moreover, it provides a toolkit for a systematic exploitation of SAPNs in bioengineering in the context of vaccine design, predicting the density of B-cell epitopes on the SAPN surface, which is critical for a strong humoral immune response.

## 1. Introduction

A promising route in the fight against major disease, such as malaria [1,2], SARS [3], influenza [4], HIV [5] and toxoplasmosis [6], is a novel family of nanoparticle-based vaccines [7,8]. They rely on a special class of self-assembling protein nanoparticles (called SAPNs) that form from multiple copies of a purpose-designed protein chain, functionalized to present epitope antigens on the particle surface. Other approaches to design protein-based nanoparticulate systems have been published by various research groups [9,10]. The architecture of such designs have been described with high accuracy [11,12]. A major challenge in the rational design of such SAPNs lies in the control of their surface structures, as building blocks can self-assemble into a spectrum of different particle morphologies. Starting with the work of Raman *et al.* [13], several SAPN species have been synthesized, but their structures have not been completely determined in most cases, and nanoparticle populations are usually characterized in terms of the diameter of the particles only. In some studies, the numbers of the protein chains composing the particle have been identified. For example, Kaba *et al.* [1] and Raman *et al.* [13] report particles corresponding to assemblies of 60 chains; Pimentel *et al.* [3] describe SAPNs with 120 chains; Yang *et al.* [14] discuss species made of 180 and 300 chains; and finally, Indelicato *et al.* [15] report assemblies of 240, 300, 360 chains. Also smaller assemblies, so-called LCM units containing 15 protein chains have been discussed and reported [13,16]. However, an exhaustive enumeration of all possible nanoparticle morphologies that can arise from multiple copies of a given type of building block is currently lacking. This presents a bottleneck in the prediction of the display of B-cell epitopes on the surface of the SAPNs to render them optimal repetitive antigen display systems.

The challenge of enumerating all possible SAPN geometries is reminiscent of the one faced in the classification of virus structures. Similar to SAPNs, viruses assemble the protein containers that encapsulate their genomes (viral capsids) from multiple copies of a small number of different capsid proteins, in many cases a single type of capsid protein. These proteins typically group together in clusters of two, three, five or six in the capsid surface, akin to the clusters seen in SAPN architectures. Caspar & Klug’s seminal classification scheme of viral architectures [17] relies on a geometric approach, predicting the spectrum of possible virus architectures in terms of the numbers and relative positions of these protein clusters (capsomeres) with reference to spherical surface lattices. This classification has revolutionized our understanding of virus structure, and plays a key role in the interpretation of experimental data in virology. This classification of virus architectures has been developed for particles with icosahedral symmetry and, as such, can be used also for synthetic vaccines based on virus-like particles, but is not suitable to model SAPNs.

We develop here a classification scheme for SAPN morphologies in terms of surface tessellations and associated graphs that pinpoint the positions of the protein building blocks in the particle surfaces. Our approach exploits the geometric relation of SAPN morphologies with fullerene architecture, and further develops tools that have been introduced for fullerene classification. As a result, we present a procedure to classify SAPN morphologies both symmetric and asymmetric, and we deliver a classification for high and low symmetry particles seen in the experiments. In particular, we explicitly determine particle morphologies for symmetric particles formed from up to 360 protein building blocks, as there is experimental evidence that spherical particles up to this size should exist, and these are relevant for vaccine design [1,3,14,15]. Defective nanoparticles are not considered in this work as they require a different mathematical model, and will be the object of future investigation.

## 2. Self-assembling protein nanoparticle morphologies and their mathematical representation as spherical graphs

**Figure 1.**
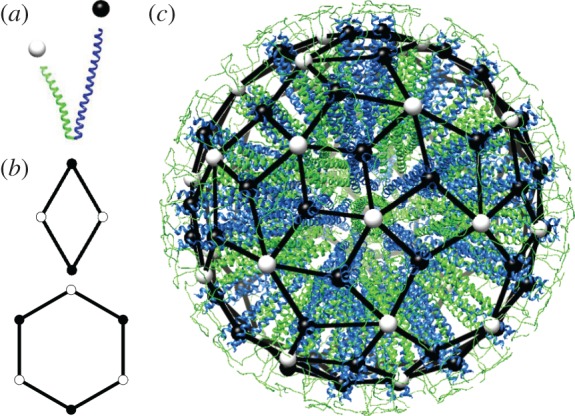
SAPN architecture and nanoparticle graphs. (*a*) The SAPN building blocks consist of two fused polypeptide helices, that cluster in groups of three (black sphere) and five (white sphere) in the nanoparticle shell. (*b*) Nanoparticle graphs correspond to spherical tessellations in terms of rhombs and hexagons, with vertices labelled alternatingly by black and white spheres. (*c*) A SAPN formed from 180 PBBs, together with its nanoparticle graph (adapted from a figure by N. Wahome and P. Burkhard). The nanoparticle model was built using a variety of adapted tools from the CCP4 program suite (www.ccp4.ac.uk/), the modelling software O (xray.bmc.uu.se/) and data from the RCSB database (www.rcsb.org/). The nanoparticle graph has been obtained by modifying a fullerene graph of the library of the Fullerene Program [18].

SAPNs are formed from multiple copies of a single protein building block (PBB) that is designed to self-assemble into particles via formation of specific cluster types. We focus here on SAPNs used in vaccine design, with PBBs given by pairs of linked helices (figure 1*a*). These are designed to interact via formation of trimeric and pentameric coiled coils involving, respectively, three (blue) and five (green) helices of different PBBs. SAPN architectures are thus characterized by the numbers and positions of these threefold and fivefold clusters.

As the trimeric and pentameric coiled coils are connected in the PBBs, SAPNs can be represented as spherical graphs in which vertices mark trimer (black spheres in figure 1) and pentamer (white spheres) positions, and edges represent the PBBs connecting them. We refer to these graphs as *nanoparticle graphs*. In vaccine design, the PBB helices are functionalized, e.g. via an extension of the trimer-forming helices by viral epitopes as in the case of the SARS HRC1 [3]. Information on the positions of the trimeric coiled coils therefore provides insights into epitope location in the nanoparticle surface. For example, figure 1*c* illustrates how nanoparticle graphs translate into SAPN morphologies, based on the example of a particle formed from 180 PBBs. It has 36 pentameric and 60 trimeric clusters, with epitope positions marked by black spheres. A classification of nanoparticle graphs thus provides an atlas of SAPN geometries and epitope positions.

## 3. Nanoparticle graphs as tilings

By construction, nanoparticle graphs have two types of vertices, *V*_3_ and *V*_5_, in which, respectively, precisely three or five edges meet. From a mathematical point of view, they are bipartite, (3,5)-regular spherical graphs. Such graphs can be viewed as spherical surface tessellations (tilings) in terms of shapes that have an even number of edges connecting, alternatingly, vertices from *V*_3_ and *V*_5_. For the sake of simplicity, we focus our analysis on tessellations in terms of hexagons and rhombs (i.e. the shapes with the smallest number of edges) with edges alternatingly marked via black and white spheres along their boundaries (figure 1*b*).

As each PBB corresponds to an edge in the nanoparticle graph, connecting a trimeric coiled coil (a vertex from *V*_3_) with a pentameric coiled coil (a vertex from *V*_5_), the number *N* of its edges must satisfy *N*=3|*V*_3_|=5|*V*_5_|. This results in the restriction

$$N=15m,\;|V_{3}|=5m,\;|V_{5}|=3m,$$

with m∈N, implying that the number of PBBs in any particle must be a multiple of 15.

For a nanoparticle graph with *N*=15*m* chains, Euler’s formula *f*=2−*v*+*e* relates the numbers of vertices *v*=|*V*_3_|+|*V*_5_|=8*m*, edges *e* and faces *f* of the corresponding spherical tiling. Using the fact that edges fulfil the condition 4*r*+6*x*=2*e*=2*N*=30*m*, with *r* and *x* denoting the number of rhombs and hexagons, respectively, one obtains

r=6(m+1),x=m−4.

As the number of hexagons must be zero or larger, this implies *m*≥4, and the nanoparticle with *N*=60 is thus the smallest possible option. Its nanoparticle graph corresponds to a rhombic triacontahedron, i.e. an icosahedrally symmetric polyhedron with 30 rhombic faces, 60 edges, 12 fivefold vertices, and 20 threefold vertices.

## 4. Nanoparticles and fullerenes

An exhaustive enumeration of nanoparticle graphs is a combinatorial challenge. We introduce here a method that relates SAPN geometries with those of fullerene cages, i.e. three-coordinated cages with vertices formed from carbon atoms. From a mathematical point of view, fullerenes correspond to three-regular spherical graphs with 12 pentagonal and otherwise hexagonal faces, and their geometries have been classified previously [18–20]. Using the method presented below, this classification of fullerene graphs can be used to derive a classification of SAPNs in terms of nanoparticle graphs.

**Figure 2.**
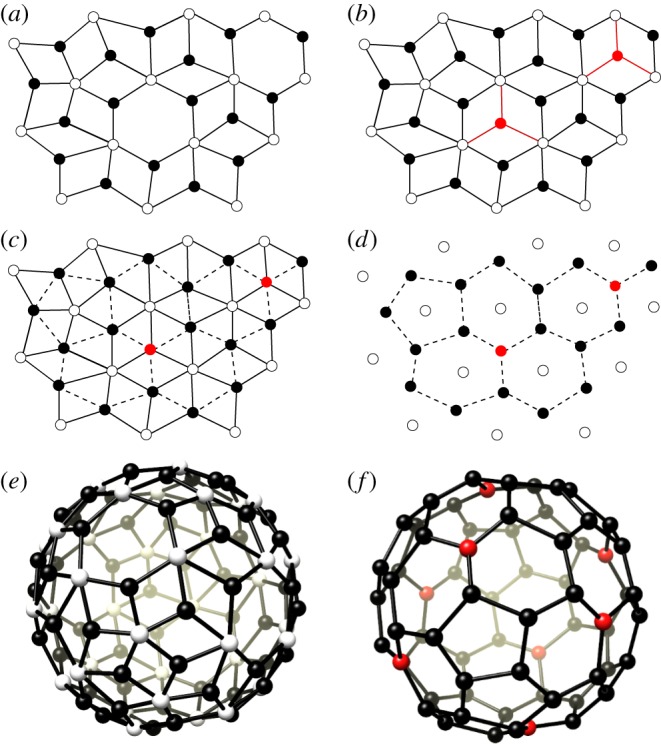
The vertex addition rule for the construction of the fullerene equivalent of a nanoparticle graph. (*a*) A portion of a nanoparticle graph; (*b*) the vertex addition rule adds additional three-coordinated vertices (red) at the centres of the hexagonal faces with connections to the five-coordinated vertices (white) of the nanoparticle graph; (*c*) pairs of trimers belonging to the same rhomb are connected by a dashed line; (*d*) removal of all edges of the nanoparticle graph in (*a*) results in a fullerene graph; (*e*) the nanoparticle graph for a particle with *N*=180 PBBs; and (*f*) the fullerene graph *C*_68_ (*T*_*d*_) obtained via the vertex addition rule, with red points representing the trimers added to the nanoparticle graph in the procedure.

**From nanoparticles to fullerenes**. To any nanoparticle graph N with isolated hexagons, i.e. in which hexagonal tiles do not share a vertex, a *unique* fullerene graph F can be associated via the following *vertex addition rule* (figure 2). In step one, a trimer is added at the centre of every hexagonal face and is connected to the white vertices (pentamers) on its boundary, resulting in a tessellation in terms of rhombs (graph $N^{\prime }$). In step two, every pair of black vertices (trimers) on the boundary of the same rhomb is connected along a diagonal of the rhomb. In step three, vertices from *V*_5_ (white) and all edges of $N^{\prime }$ are removed. The remaining vertices *V* ′_3_, given by the union of *V*_3_ (black vertices) and the (red) vertices added in step one, and their connections via the edges added in step two, define the fullerene graph F. The vertex addition rule relates the number of vertices, edges and faces of a nanoparticle graph with that of its fullerene graph counterpart according to table 1.

**Table 1.** Relationship between a nanoparticle graph and its associated fullerene graph.

	nanoparticle N	nanoparticle $N^{\prime }$	fullerene F
edges	15*m*	18*m*−12	9*m*−6
degree-3 vertices	5*m*	6*m*−4	6*m*−4
degree-5 vertices	3*m*	12	
degree-6 vertices		3*m*−12	
rhombic faces	6(*m*+1)	9*m*−6	
hexagonal faces	*m*−4		3*m*−12
pentagonal faces			12

**From fullerenes to nanoparticles**. The above procedure is not always reversible. Reversal would require completion of the following three steps. In step one, the set *V*_5_ of the nanoparticle graph is constructed by placing a vertex at the centre of each face of the fullerene graph F, i.e. by adding the vertices of the dual graph of F to the vertices *V* ′_3_ of the fullerene graph. In step two, each such vertex is connected to those vertices from *V* ′_3_ that are located on the same face, and all edges of the fullerene graph are removed. This yields a bipartite graph $N^{\prime }$ with vertices of degree 3 (*V* ′_3_) and vertices of degree either 5 or 6 (*V*_5_). Finally, in order to obtain a nanoparticle graph N, removal of vertices from *V* ′_3_ is required so that all vertices in *V*_5_ have degree 5. This requires eliminating (colouring) of exactly one vertex of the fullerene graph F for each hexagonal face, and none from the pentagonal faces, which we will refer to as the *vertex colouring rule* in the following. Such colouring may not be possible or not be unique. A necessary condition for a fullerene graph to result in a nanoparticle graph with *N*=15*m* edges via the vertex colouring rule is that the fullerene graphs must have 6*m*−4 vertices, corresponding to the sum of the number of vertices *V*_3_ and hexagonal faces *x* of the nanoparticle graph. We will therefore in the following classify SAPN morphologies, either symmetric or not, starting with fullerene graphs *C*_6*m*−4_, that have been classified previously [18–20].

## 5. Results

Fullerene cages can have varying degrees of symmetry, including the icosahedral symmetry of the Buckminster fullerene and carbon onions, the lower dihedral symmetries of prolate architectures, and the asymmetric shapes of fullerene cones. Similarly, nanoparticle graphs and SAPNs can vary in symmetry. We start with a classification of nanoparticle graphs with non-planar symmetries, i.e. those with at least two different types of symmetry axes. Note that fourfold symmetry axes cannot occur. This is because vertices of nanoparticle graphs cannot occupy fourfold axes, and octagonal faces are excluded. Therefore, icosahedral and tetrahedral symmetries are the only possible non-planar options.

Symmetry imposes strong restrictions on the number *N* of edges of the nanoparticle graph, so that only particles with certain numbers of PBBs are allowed. In order to construct the nanoparticle graphs for these cases explicitly, we adapt methods used previously in the context of fullerene architecture. In particular, for the modelling of the icosahedrally symmetric nanoparticle graphs we adapt the Goldberg–Coxeter procedure [21,22], and for the tetrahedral graphs we use its extension to tetrahedral symmetry by Fowler *et al.* [23]. In each case, we first construct the fullerenes with required symmetry and number of edges, and then derive the corresponding nanoparticle graphs via the vertex colouring rule in figure 2.

### 5.1. Icosahedral nanoparticles

We first derive restrictions owing to symmetry. Consider the icosahedral group *I* acting on the nanoparticle graph (embedded into a sphere). Denote by *t*_*d*_ and *p*_*d*_ the number of trimers and pentamers in generic positions in the fundamental domain, i.e. those not positioned on symmetry axes of the particle. Then, for the particle to have icosahedral symmetry, the following relationship has to be fulfilled:

$$N=3\underset{\begin{array}{c} \mathrm{total}\;\mathrm{number}\\ \mathrm{of}\;\mathrm{trimers} \end{array}}{\underbrace{\left(20\alpha +60t_{d}\right)}}=5\underset{\begin{array}{c} \mathrm{total}\;\mathrm{number}\\ \mathrm{of}\;\mathrm{pentamers} \end{array}}{\underbrace{\left(12\gamma +60p_{d}\right)}}.$$

Here, *α*=1 or 0 indicates the presence or absence of trimers on the threefold axes of icosahedral symmetry, and *γ*=1 or 0 of pentamers on the fivefold axes, respectively. Note that, as *I* is a subgroup of the full icosahedral group *I*_*h*_, this restriction also holds for nanoparticles with full icosahedral symmetry. There are only two solutions up to *N*=360, given by *N*=60 and *N*=360.

**Figure 3.**
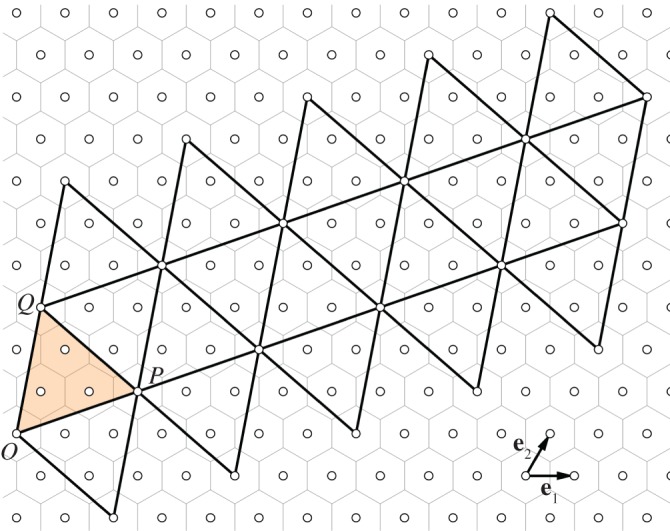
The Goldberg–Coxeter construction for particles with icosahedral symmetry. A superposition of an icosahedral net on a hexagonal tessellation determines the positions of hexagonal and pentagonal faces in a fullerene. The example shows the construction for a particle with (*i*,*j*)=(2,1), where *O*=(0,0), *P*=(2,1) and *Q*=(−1,3) with respect to the triangular lattice basis (**e**_1_,**e**_2_).

We use the Goldberg–Coxeter construction for fullerenes to determine the corresponding nanoparticle graphs. In this construction, a fullerene graph is represented as a superposition of an icosahedral surface (20 equilateral triangular faces) on a planar hexagonal grid such that the icosahedral vertices coincide with centres of the hexagonal tiles (figure 3). The positions of the carbon atoms in the fullerene then correspond to the vertices of the hexagonal tiles that overlap with the embedded icosahedral surface. Denoting one of the icosahedral vertices as *O*, the construction is fully determined by specifying the location of a second vertex *P* on the same triangular face in terms of integer coordinates (*i*,*j*) in the hexagonal lattice basis **e**_1_ and **e**_2_. The equilateral triangle with *P*=(1,0) by construction contains only one vertex of the fullerene graph, i.e. one carbon atom. Denoting the area of this triangle as *Δ*, then an equilateral triangle with vertices at (0,0) and *P*=(*i*,*j*) has area (*i*^2^+*ij*+*j*^2^)*Δ*, and therefore contains *i*^2^+*ij*+*j*^2^ vertices of the fullerene. Given that the planar net of the icosahedron contains 20 equilateral triangular faces, fullerenes with icosahedral symmetry are only possible if they have 20(*i*^2^+*ij*+*j*^2^) vertices. As only fullenene graphs with 6*m*−4 vertices can correspond to nanoparticle graphs with *N*=15*m* chains (recall table 1), we obtain the condition

$$i^{2}+ij+j^{2}=\frac{1}{50}(N-10).$$

The two possible solutions *N*=60 and *N*=360 correspond to isomers with (*i*,*j*)=(1,0) or (*i*,*j*)=(0,1), and (*i*,*j*)=(2,1) or (*i*,*j*)=(1,2), respectively. In each case, we construct the planar net and apply the vertex colouring rule. In the first case, the nanoparticle graph has no hexagons and corresponds to the rhombic triacontahedron. In the second case, colouring compatible with icosahedral symmetry is indeed possible and results in two structures that are identical up to helicity (cf. table 2).

**Table 2.** Nanoparticles with non-planar symmetries. (Data on fullerenes in this table are excerpts from the Fowler–Cremona–Steer classification.)

*N*	fullerene	fullerene symmetry	(*i*,*j*,*h*,*k*)	nanoparticle symmetry
60	*C*_20_	*I*_*h*_	(1,0,0,1)	*I*_*h*_
120	*C*_44_	*T*	(2,0,0,1)	*T*
180	*C*_68_	*T*	(1,2,−1,1)	—
		*T*_*d*_	(1,2,0,2)	*T*_*d*_
240	*C*_92_	*T*	(2,1,0,2)	*T*
		*T*_*h*_	(2,1,1,2)	*T*_*h*_
		*T*_*d*_	(3,1,0,1)	*T*,*T* (chiral)
300	*C*_116_	*T*	(1,2,−2,2)	*T*, *T*
		*T*_*h*_	(1,2,−1,3)	*T*,*T* (chiral)
		*T*	(4,0,0,1)	*T*
360	*C*_140_	*I*	(1,2,−2,3)	*I*
		*T*	(2,3,−1,1)	—
		*T*	(3,1,0,2)	—
		*T*	(3,1,1,2)	—

### 5.2. Tetrahedral nanoparticles

**Figure 4.**
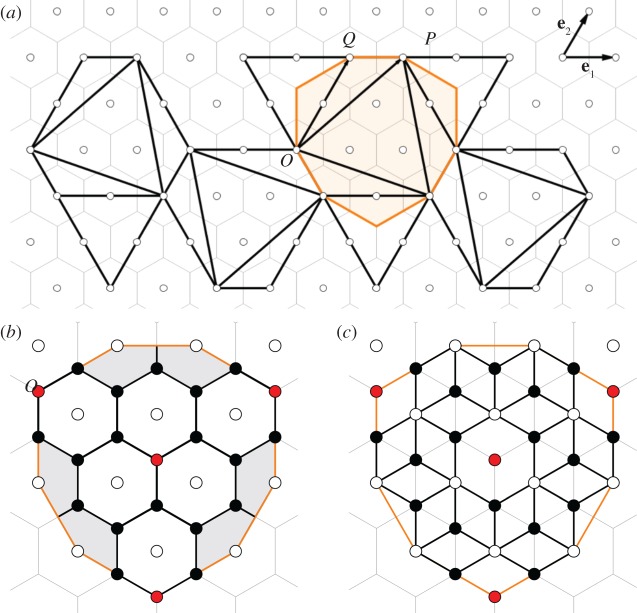
(*a*) The Fowler–Cremona–Steer construction for fullerenes with tetrahedral symmetry. Here *O*=(0,0), *P*=(1,2), *Q*=(0,2) with respect to the triangular lattice basis, so that (*i*,*j*)=(1,2) and (*h*,*k*)=(0,2). The domain used here for the construction of the nanoparticles (corresponding in area to three times the fundamental domain) is shown highlighted; (*b*) a close up at this domain for the case of fullerene *C*_68_, with areas corresponding to portions of six of the 12 pentagons of the fullerene shown in grey; and (*c*) close-up of the domain in the corresponding nanoparticle graph (*N*=180), with trimers deleted according to the vertex colouring rule shown in red.

As before, we first derive symmetry restrictions on *N*. Denoting by *t*_*d*_ and *p*_*d*_ the number of trimers and pentamers in generic position in the fundamental domain, the symmetry condition is

$$N=3(4\alpha +4\beta +12t_{d})=5(12p_{d}),$$

where *α*, *β* in {0,1} indicate the absence or presence of trimeric clusters on the two types of threefold sites. Note that these correspond, respectively, to corners and centres of faces of a tetrahedron. The solutions specify the allowed chain numbers for particles with tetrahedral symmetry. Up to and including 360 chains, these are *N*=60 for (*α*,*β*,*t*_*d*_,*p*_*d*_)=(1,1,1,1); *N*=120 for (0,1,3,2) and (1,0,3,2); *N*=180 for (0,0,5,3); *N*=240 for (1,1,6,4); *N*=300 for (0,1,8,5) and for (1,0,8,5); and *N*=360 for (0,0,10,6). By table 1, these correspond to fullerenes *C*_*n*_ with *n*=20,44,68,92,116,140. Note also that, because *T* is a subgroup of the tetrahedral groups *T*_*h*_ and *T*_*d*_, the above restrictions hold also for nanoparticles with higher tetrahedral symmetry. Fullerenes with tetrahedral symmetry can be constructed via the Fowler–Cremona–Steer construction [23], which is based on the superposition of the surface of a polyhedron with tetrahedral symmetry onto a planar hexagonal tessellation as shown in figure 4. The polyhedral surface corresponds to the union of three types of triangles, equilateral and scalene, which are characterized via a quadruplet of integers (*i*,*j*,*h*,*k*) as follows: the four *large* equilateral triangles are given as in the Goldberg–Coxeter construction via (*i*,*j*), and the four *small* equilateral triangles by (*h*,*k*) (points *P* and *Q* in figure 4); the 12 scalene triangles are then implied by the dimensions and positions of these two triangle types. If all edges are of the same length and the angles between the large and small equilateral triangles are 60^°^ (which is the case for *h*=−*j* and *k*=*i*+*j*), then this construction results in an icosahedral net as in figure 3. In general, the area of the polyhedral surface is 4(*i*^2^+*ij*+*j*^2^+*h*^2^+*hk*+*k*^2^+3(*ik*−*jh*)) times the area of a small equilateral triangle with vertices at (0,0) and (1,0). We thus obtain, using table 1, that the corresponding nanoparticle graph with *N* edges must satisfy the identity

$$N=10+10(i^{2}+ij+j^{2}+h^{2}+hk+k^{2}+3(ik-jh)).$$

**Figure 5.**
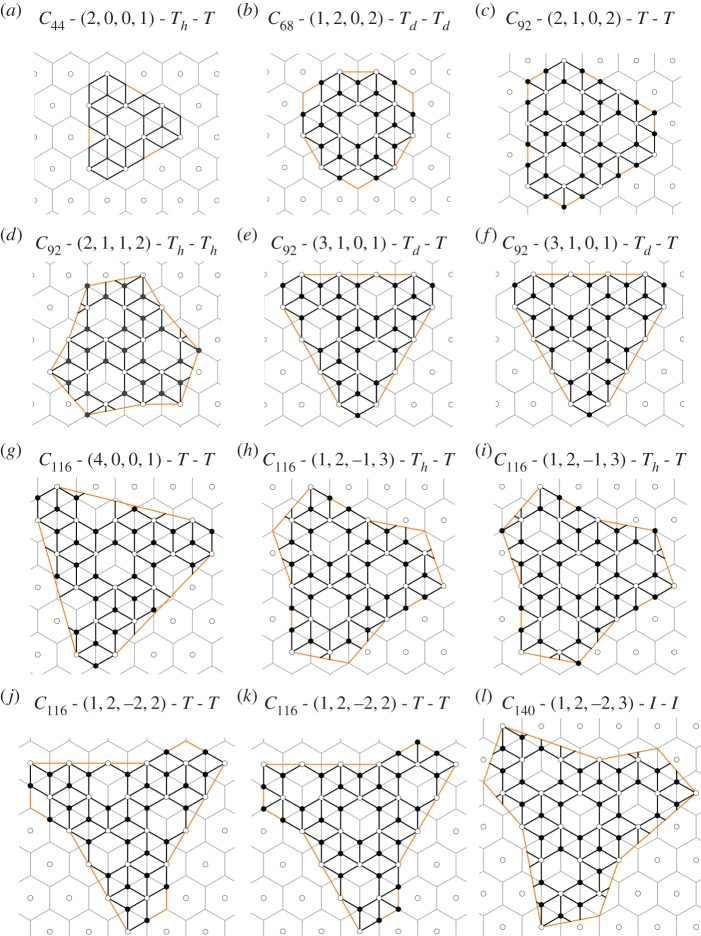
Atlas of tetrahedral and icosahedral nanoparticles; the depicted domains are the union of three fundamental domains of the tetrahedral group (cf. figure 4).

We construct the planar nets for all tetrahedral solutions above, using the (*i*,*j*,*h*,*k*) vectors from the Fowler–Cremona–Steer classification (table 2), and check whether the vertex colouring procedure can be applied to obtain a nanoparticle graph. Note that the colouring is not always possible, and that there are cases in which there are different nanoparticles corresponding to the same fullerene. We list all resulting nanoparticle graphs with at least tetrahedral symmetry in table 2 and provide the corresponding atlas in figure 5. We give an explicit example of the full net of a tetrahedral fullerene graph and its associated nanoparticle graph (electronic supplementary material, figures S1 and S2).

### 5.3. Particles with lower symmetry

The procedure introduced above allows one to construct nanoparticle graphs with arbitrary, or lack of, symmetry. In particular, as nanoparticle graphs with rhombic and hexagonal faces cannot have sixfold axes, neither sixfold rotational symmetry axes nor *D*_6_ symmetry are possible. By contrast, particles with *D*_5_ and *D*_3_ symmetry can occur.

Particles with *D*_5_ symmetry must fulfil the necessary condition

$$N=5(2\alpha +10p_{d})=3(10t_{d}),$$

where *α*=1 when the two sites of fivefold symmetry are each occupied by pentamers, and *p*_*d*_,*t*_*d*_ have the same meaning as before. Note that the exclusion of decagonal tiles implies *α*=1. There are only three possible solutions for chain numbers up to and including 360: *N*=60 (and *C*_20_) for (*p*_*d*_,*t*_*d*_,*m*)=(1,2,4); *N*=210 (and *C*_80_) for (*p*_*d*_,*t*_*d*_,*m*)=(4,7,14); and *N*=360 (and *C*_140_) for (*p*_*d*_,*t*_*d*_,*m*)=(7,12,24). As before, models of fullerenes with *D*_5_ symmetry can be constructed by superimposing the general planar net of a polyhedron with such symmetry onto a hexagonal tessellation of the plane (cf. [23]). This again requires the specification of four integers (*i*,*j*,*h*,*k*), and corresponding values are listed in table 3.

**Table 3.** Nanoparticles derived from fullerene graphs with *D*_5_ symmetry.

*N*	fullerene	fullerene symmetry	(*i*,*j*,*h*,*k*)	nanoparticle symmetry
60	*C*_20_	*I*_*h*_	(1,−1,1,0)	*I*_*h*_
210	*C*_80_	*D*_5*d*_	(4,−7,1,0)	—
		*D*_5*d*_	(3,−2,1,1)	—
		*I*_*h*_	(2,−2,2,0)	—
		*D*_5*h*_	(1,−2,2,0)	—
		*D*_5*h*_	(1,0,2,1)	*D*_5_, *D*_5_
360	*C*_140_	*D*_5*d*_	(7,−13,1,0)	—
		*D*_5*d*_	(6,−5,1,1)	—
		*I*	(3,−1,1,2)	*I*, *D*_5_
		*D*_5_	(3,−5,2,0)	—
		*D*_5_	(1,0,2,2)	—
		*D*_5_	(1,0,3,1)	*D*_5_, *D*_5_

Note that the nanoparticle corresponding to *C*_20_ yields the classical icosahedral solution, while the isomer of *C*_80_ with coordinates (1,0,2,1) results in two different particles with *D*_5_ symmetry. Just two isomers of *C*_140_ yield solutions upon colouring (three of which have *D*_5_ symmetry and one *I*). All colourings generating nanoparticles with at least *D*_5_ symmetry are listed in table 3.

Regarding *D*_3_ symmetry, the necessary condition is

$$N=5(6p_{d})=3(6t_{d}+2\alpha ),$$

where *α*∈{0,1} indicates the absence or presence of trimeric clusters on the particle threefold axes. Inspection of the Fowler–Cremona–Steer construction shows that the two sites of threefold symmetry must both be occupied by trimers, so that *α*=1. There are four solutions for particles up to 360 chains as follows: *N*=60 (*C*_20_) with (*p*_*d*_,*t*_*d*_,*m*)=(2,3,4); *N*=150 (*C*_56_) with (*p*_*d*_,*t*_*d*_,*m*)=(5,8,10); *N*=240 (*C*_92_) with (*p*_*d*_,*t*_*d*_,*m*)=(8,13,16); and *N*=330 (*C*_128_) with (*p*_*d*_,*t*_*d*_,*m*)=(11,18,22). The general planar net of a polyhedron with *D*_3_ symmetry can be represented as the union of four types of triangles, equilateral and scalene, which require the specification of six integers of the form (0,1,1,0,0,*n*′) with *n*′≥1 [23]. Values for nanoparticles corresponding to the *D*_3_ solutions are listed in table 4.

**Table 4.** Nanoparticles derived from fullerene graphs with *D*_3_ symmetry.

*N*	fullerene	fullerene symmetry	(0,1,1,0,0,*n*′)	nanoparticle symmetry
60	*C*_20_	*I*_*h*_	(0,1,1,0,0,1)	*I*_*h*_
150	*C*_56_	*D*_3*d*_	(0,1,1,0,0,7)	*D*_3_, *D*_3_
240	*C*_92_	*D*_3*d*_	(0,1,1,0,0,13)	4 solutions *D*_3_
330	*C*_128_	*D*_3*d*_	(0,1,1,0,0,19)	8 solutions *D*_3_

Nanoparticles with lower symmetry can also occur in all of these cases if the vertex colouring rule is applied in such a way to the associated fullerene graphs *C*_6*m*−4_ that its symmetry is reduced or broken. An example of this is provided in the electronic supplementary material, figure S3, showing all ways in which the symmetry of the icosahedral particle with 360 chains can be reduced. This demonstrates how the procedure developed here can be used to determine all lower symmetry alternatives for any of the higher symmetry particles listed in the previous subsection.

## 6. Exploitation in the context of vaccine design

These results pave the way for the optimization of SAPN morphologies for applications in vaccine design. To generate an optimal humoral immune response, repetitive antigen display is a key determinant [24–32]. SAPNs represent an ideal model for repetitive antigen display. They are similar to virus-like particles, but they have the advantage that they are more flexible in protein design, allowing testing of different architectures relatively easily. B-cell epitopes can be attached to either end of the protein chain and will thus be displayed close to the trimer and pentamer vertices of the particular SAPN architecture.

The geometries as outlined above allow straightforward calculation of the distances between epitopes. This defines the epitope density, which in turn is related to the strength of the immune response. Already several decades ago, in their hallmark publication Dintzis *et al.* [33] related the epitope density to the so-called immunon, a determinant of the strength of the immune response. Based on our results, we can estimate the average distance between trimers and pentamers by a simple density argument. Given a nanoparticle graph N, with *N*=15*m* edges, consider the associated graph $N^{\prime }$, in which each hexagon is replaced by three rhombs (figure 2), so that $N^{\prime }$ has only rhombic faces, specifically 9*m*−6 (by table 1). Assuming that all rhombs are approximately equal, with approximately the same area, shape and sides, the area *A* of a spherical rhomb on the surface of a sphere of radius *r* can be estimated as

$$A∼\frac{4\pi r^{2}}{9m-6}.$$

Given the area of the rhomb, and using spherical geometry we obtain table 5 for the average distances between trimers and between pentamers on a sphere of radius *r*.

**Table 5.** The average distances between trimers and pentamers on a sphere of radius *r*.

*N*=15*m*	average distance between trimers	average distance between pentamers
60	0.7136 *r*	1.0514 *r*
120	0.5269 *r*	0.7607 *r*
150	0.4763 *r*	0.6834 *r*
180	0.4381 *r*	0.6257 *r*
210	0.4078 *r*	0.5805 *r*
240	0.3830 *r*	0.5439 *r*
300	0.3446 *r*	0.4875 *r*
330	0.3293 *r*	0.4652 *r*
360	0.3159 *r*	0.4457 *r*

The epitopes can be on either end of the SAPN, i.e. on the trimer or on the pentamer. Identical epitopes will however always be on the same oligomerization domain. Computer modelling and experimental analysis have shown that the radius of the central cavity of the SAPNs, i.e. where the two coiled coils are joined together for a SAPN with *N*=60 is about 3 nm. The dimension of the central cavity will increase with the number of protein chains per particle. Also, the B-cell epitope will not be located on top of the vertices but rather roughly on top of the individual *α*-helical axes. The distance of this axis of the coiled coil *α*-helices relative to the trimer and pentamer axes is about 0.65 nm and 0.85 nm for the trimer and pentamer, respectively [34,35]. These two values have to be subtracted from the calculated distance between either two trimer or two pentamer vertices in table 5.

If the B-cell epitope itself is a coiled-coil trimer as for example in the SARS [3] vaccines then we can calculate the distance between adjacent B-cell epitopes for a given length of a coiled coil. For instance, in the SARS nanoparticle with *N*=120 and a helix length of about 7 nm, the distance between epitopes located at trimeric sites would be about 4.6 nm. If the B-cell epitope itself is not coiled-coil, which has a quite extended shape, then the particular dimension of the B-cell epitope will also have to be taken into consideration. If it is a folded protein domain then it has quite likely a roughly spherical shape. The size of a protein like lysozyme is about 3.5 nm. Using a particular SAPN architecture the B-cell epitope can then be placed in an array with a rather precise spacing depending on the lengths of the coiled coil of the SAPN. This gives the vaccinologist a tool to optimize the vaccine for best immune response.

## 7. Discussion

The classification presented here provides, to our knowledge, the first complete atlas of SAPN geometries of *D*_3_ symmetry or higher, and provides a construction method for all particles, including low symmetry and asymmetric ones. We have demonstrated previously that a combinatorial analysis of SAPN structures can be an invaluable tool in the interpretation of experimental data. In particular, biophysical methods such as analytical ultracentrifugation can provide information on the numbers of chains *N* in the particles that occur in the self-assembly process. Combinatorics does then narrow down the spectrum of options to a limited ensemble of particle geometries compatible with this range of chain numbers, and identifies the precise surface structures of the particles in terms of the placements of all protein chains and threefold and fivefold coiled coils. It also offers a glimpse at the complexity of the assembly process in terms of the numbers of different particles that can occur in a given range of chain numbers. In previous work [15], a full classification had not yet been available. It was therefore only possible to identify possible candidates for the particles seen in experiment, but an exhaustive enumeration was not possible.

The construction method with reference to fullerene architecture introduced here provides a step change. It offers for the first time, to our knowledge, insights into the full spectrum of particles of arbitrary size and morphology occurring in an experiment. This exhaustive approach therefore opens up opportunities for the analysis of experimental data that had not been possible before. For example, it is now possible to apply statistical mechanics approaches and construct partition functions describing the outcome of the assembly experiments. These can be used to better understand the assembly process itself in terms of the most likely, dominant assembly pathways. This, in turn, will provide pointers for experimentalists on how to optimize the assembly procedure, e.g. in terms of the yield of desired particle types. The detailed insights into the connectivity of each chain in the nanoparticle surface moreover enable computer reconstructions of the nanoparticles, as in the example in figure 1*c*. These can then be used to engineer specific architectures by controlling the rigidity of the links and the angle between the coiled coils (an issue not addressed here).

Most importantly, however, the results obtained here enable the identification of SAPN morphologies that have not yet been synthesized, and thus enable the rational design of desired particle morphologies. In particular, our approach links SAPN morphologies with epitope positions, and therefore provides a tool for the identification of SAPN morphologies with optimal properties for vaccine design. However, if the SAPNs are co-assembled from different chains, i.e. if the SAPNs are composed of epitope-decorated units and protein chains lacking epitopes, then the assembly forms will be much more difficult to predict. Depending on the B-cell epitope, chains with epitope may cluster together if there are attracting forces between the B-cell epitopes. Also, we do not exclude the possibility that SAPNs may be formed that have an irregular assembly form of protein chains owing to imperfect propagation of the lattice in all directions. If so, this would lead to chimeric forms of SAPNs with respect to their architecture as described here.

## Supplementary Material

Models of fullerene and nanoparticle graphs

## Data accessibility

The paper is a theoretical study and is self-contained.

## References

[1] Kaba SA *et al.* 2012 Protective antibody and CD8+ T-cell responses to the *Plasmodium falciparum* circumsporozoite protein induced by a nanoparticle vaccine. *PLoS ONE* 7, e48304 (doi:10.1371/journal.pone.0048304) 23144750 10.1371/journal.pone.0048304 PMC3483151

[2] Kaba SA, Brando C, Guo Q, Mittelholzer C, Raman SK, Tropel D, Aebi U, Burkhard P, Lanar DE 2009 A non-adjuvanted polypeptide nanoparticle vaccine confers long-lasting protection against rodent malaria. *J. Immunol.* 183, 7268–7277. (doi:10.4049/jimmunol.0901957) 19915055 10.4049/jimmunol.0901957 PMC4528972

[3] Pimentel TA, Yan Z, Jeffers SA, Holmes KV, Hodges RS, Burkhard P 2009 Peptide nanoparticles as novel immunogens: design and analysis of a prototypic severe acute respiratory syndrome vaccine. *Chem. Biol. Drug Des.* 73, 53–61. (doi:10.1111/j.1747-0285.2008.00746.x) 19152635 10.1111/j.1747-0285.2008.00746.x PMC2756483

[4] Babapoor S, Neef T, Mittelholzer C, Girshick T, Garmendia A, Shang H, Khan MI, Burkhard P 2011 A novel vaccine using nanoparticle platform to present immunogenic M2e against avian influenza infection. *Influenza Res. Treat* 2011, 126794–126805. (doi:10.1155/2011/126794) 23074652 10.1155/2011/126794 PMC3447297

[5] Wahome N, Pfeiffer T, Ambiel I, Yang Y, Keppler OT, Bosch V, Burkhard P 2012 Conformation-specific display of 4E10 and 2F5 epitopes on self-assembling protein nanoparticles as a potential HIV vaccine. *Chem. Biol. Drug Des.* 80, 349–357. (doi:10.1111/j.1747-0285.2012.01423.x) 22650354 10.1111/j.1747-0285.2012.01423.x

[6] El-Bissati K, Zhou Y, Dasgupta D, Cobb D, Dubey JP, Burkhard P, Lanar DE, Mcleod R 2014 Effectiveness of a novel immunogenic nanoparticle platform for *Toxoplasma* peptide vaccine in HLA transgenic mice. *Vaccine* 32, 3243–3248. (doi:10.1016/j.vaccine.2014.03.092) 24736000 10.1016/j.vaccine.2014.03.092 PMC4084734

[7] López-Segaseta J, Malito E, Rappuoli R, Bottomley MJ 2016 Self-assembling protein nanoparticles in the design of vaccines. *Comput. Struct. Biotechnol. J.* 14, 58–68. (doi:10.1016/j.csbj.2015.11.001) 26862374 10.1016/j.csbj.2015.11.001 PMC4706605

[8] Powles L, Xiang SD, Selomulya C, Plebanski M 2015 The use of synthetic carriers in malaria vaccine design. *Vaccines* 3, 894–929. (doi:10.3390/vaccines3040894) 26529028 10.3390/vaccines3040894 PMC4693224

[9] Sciore A *et al.* 2016 Flexible, symmetry-directed approach to assembling protein cages. *Proc. Natl Acad. Sci. USA* 113, 8681–8686. (doi:10.1073/pnas.1606013113) 27432965 10.1073/pnas.1606013113 PMC4978231

[10] Fletcher JM *et al.* 2013 Self-assembling cages from coiled-coil peptide modules. *Science* 340, 595–599. (doi:10.1126/science.1233936) 23579496 10.1126/science.1233936 PMC6485442

[11] King NP, Sheffler W, Sawaya MR, Vollmar BS, Sumida JP, André I, Gonen T, Yeates TO, Baker D 2012 Computational design of self-assembling protein nanomaterials with atomic level accuracy. *Science* 336, 1171–1174. (doi:10.1126/science.1219364) 22654060 10.1126/science.1219364 PMC4138882

[12] Padilla JE, Colovos C, Yeates TO 2001 Nanohedra: using symmetry to design self assembling protein cages, layers, crystals, and filaments. *Proc. Natl Acad Sci. USA* 98, 2217–2221. (doi:10.1073/pnas.041614998) 11226219 10.1073/pnas.041614998 PMC30118

[13] Raman SK, Machaidze G, Lustig A, Aebi U, Burkhard P 2006 Structure-based design of peptides that self-assemble into regular polyhedral nanoparticles. *Nanomedicine* 2, 95–102. (doi:10.1016/j.nano.2006.04.007) 17292121 10.1016/j.nano.2006.04.007

[14] Yang Y, Ringler P, Müller SA, Burkhard P 2012 Optimizing the refolding conditions of self-assembling polypeptide nanoparticles that serve as repetitive antigen display systems. *J. Struct. Biol.* 177, 168–176. (doi:10.1016/j.jsb.2011.11.011) 22115997 10.1016/j.jsb.2011.11.011 PMC7118850

[15] Indelicato G, Wahome N, Ringler P, Müller SA, Nieh MP, Burkhard P, Twarock R 2016 Principles governing the self-assembly of coiled-coil protein nanoparticles. *Biophys. J.* 110, 646–660. (doi:10.1016/j.bpj.2015.10.057) 26840729 10.1016/j.bpj.2015.10.057 PMC4744166

[16] Raman S, Machaidze G, Lustig A, Olivieri A, Aebi U, Burkhard P 2009 Design of peptide nanoparticles using simple protein oligomerization domains. *Open Nanomed. J.* 2, 15–26. (doi:10.2174/1875933500902010015)

[17] Caspar DL, Klug A 1962 Physical principles in the construction of regular viruses. *Cold Spring Harb. Symp. Quant. Biol.* 27, 1–24. (doi:10.1101/SQB.1962.027.001.005) 14019094 10.1101/sqb.1962.027.001.005

[18] Schwerdtfeger P, Wirz L, Avery J 2013 Program Fullerene: a software package for constructing and analyzing structures of regular fullerenes, version 4.4. *J. Comput. Chem.* 34, 1508–1526. (doi:10.1002/jcc.23278) 23559399 10.1002/jcc.23278

[19] Fowler PW, Manolopoulos DE 2006 *An atlas of fullerenes*. New York, NY: Dover.

[20] Schwerdtfeger P, Wirz L, Avery J 2015 The topology of fullerenes. *WIREs Comput. Mol. Sci.* 5, 96–145. (doi:10.1002/wcms.1207) 10.1002/wcms.1207 PMC4313690 25678935

[21] Goldberg M 1937 A class of multi-symmetric polyhedra. *Tohoku Math. J.* 43, 104–108.

[22] Coxeter HSM 1971 Virus macromolecules and geodesic domes. In *A spectrum of mathematics* (ed. JC Butcher), pp. 279–303. Oxford, UK: Oxford University Press.

[23] Fowler PW, Cremona JE, Steer JI 1988 Systematics of bonding in non-icosahedral carbon clusters. *Theor. Chim. Acta* 73, 1–26. (doi:10.1007/BF00526647)

[24] Fehr T, Bachmann MF, Bucher E, Kalinke U, Di Padova FE, Lang AB, Hengartner H, Zinkernagel AM 1997 Role of repetitive antigen patterns for induction of antibodies against antibodies. *J. Exp. Med.* 185, 1785–1792. (doi:10.1084/jem.185.10.1785) 9151704 10.1084/jem.185.10.1785 PMC2196322

[25] Bachmann MF, Kalinke U, Althage A, Freer G, Burkhart C, Roost H, Aguet M, Hengartner H, Zinkernagel RM 1997 The role of antibody concentration and avidity in antiviral protection. *Science* 276, 2024–2027. (doi:10.1126/science.276.5321.2024) 9197261 10.1126/science.276.5321.2024

[26] Baschong W, Hasler L, Häner M, Kistler J, Aebi U 2003 Repetitive versus monomeric antigen presentation: direct visualization of antibody affinity and specificity. *J. Struct. Biol.* 143, 258–262. (doi:10.1016/j.jsb.2003.08.004) 14572480 10.1016/j.jsb.2003.08.004

[27] Bachmann MF, Jennings GT 2010 Vaccine delivery: a matter of size, geometry, kinetics and molecular patterns. *Nat. Rev. Immunol.* 10, 787–796. (doi:10.1038/nri2868) 20948547 10.1038/nri2868

[28] Yassine HM *et al.* 2015 Hemagglutinin-stem nanoparticles generate heterosubtypic influenza protection. *Nat. Med.* 21, 1065–1070. (doi:10.1038/nm.3927) 26301691 10.1038/nm.3927

[29] Kanekiyo M *et al.* 2013 Self-assembling influenza nanoparticle vaccines elicit broadly neutralizing H1N1 antibodies. *Nature* 499, 102–106. (doi:10.1038/nature12202) 23698367 10.1038/nature12202 PMC8312026

[30] Jardine J *et al.* 2013 Rational HIV immunogen design to target specific germline B cell receptors. *Science* 340, 711–716. (doi:10.1126/science.1234150) 23539181 10.1126/science.1234150 PMC3689846

[31] Correia BE *et al.* 2014 Proof of principle for epitope-focused vaccine design. *Nature* 507, 201–206. (doi:10.1038/nature12966) 24499818 10.1038/nature12966 PMC4260937

[32] Brune KD, Leneghan DB, Brian IJ, Ishizuka AS, Bachmann MF, Draper SJ, Biswas S, Howarth M 2016 Plug-and-display: decoration of virus-like particles via isopeptide bonds for modular immunization. *Sci. Rep.* 6, 19234 (doi:10.1038/srep19234) 26781591 10.1038/srep19234 PMC4725971

[33] Dintzis HM, Dintzis RZ, Vogelstein B 1976 Molecular determinants of immunogenicity: the immunon model of immune response. *Proc. Natl Acad. Sci. USA* 73, 3671–3675. (doi:10.1073/pnas.73.10.3671) 62364 10.1073/pnas.73.10.3671 PMC431180

[34] Strelkov SV, Burkhard P 2002 Analysis of alpha-helical coiled coils with the program TWISTER reveals a structural mechanism for stutter compensation. *J. Struct. Biol.* 137, 54–64. (doi:10.1006/jsbi.2002.4454) 12064933 10.1006/jsbi.2002.4454

[35] Malashkevich VN, Kammerer RA, Efimov VP, Schulthess T, Engel J 1996 The crystal structure of a five-stranded coiled coil in COMP: a prototype ion channel? *Science* 274, 761–765. (doi:10.1126/science.274.5288.761) 8864111 10.1126/science.274.5288.761

