# Supplementary material for: Classification of self-assembling protein nanoparticle architectures for applications in vaccine design
Source: R Soc Open Sci. 2017 Apr 26;4(4):161092. doi: 10.1098/rsos.161092 (PMC5414263; doi:10.1098/rsos.161092)
Supplement: Models of fullerene and nanoparticle graphs [file rsos161092supp1.pdf]

Supplementary Information for "Classification of self-assembling protein nanoparticle architectures for applications in vaccine design" published in Royal Society Open Science

Giuliana Indelicato, Peter Burkhard and Reidun Twarock

Figures S1 and S2 show a full planar representation of the tetrahedral fullerene C_{68} (using the construction in Fowler PW, Cremona JE, Steer JI (1988) Systematics of Bonding in non-Icosahedral Carbon Clusters. *Theor Chim Acta* 73: 1-26.) and a nanoparticle obtained by the vertex coloring rule. The figures can be cut and folded to constuct a 3D representation of the nanoparticle graphs.

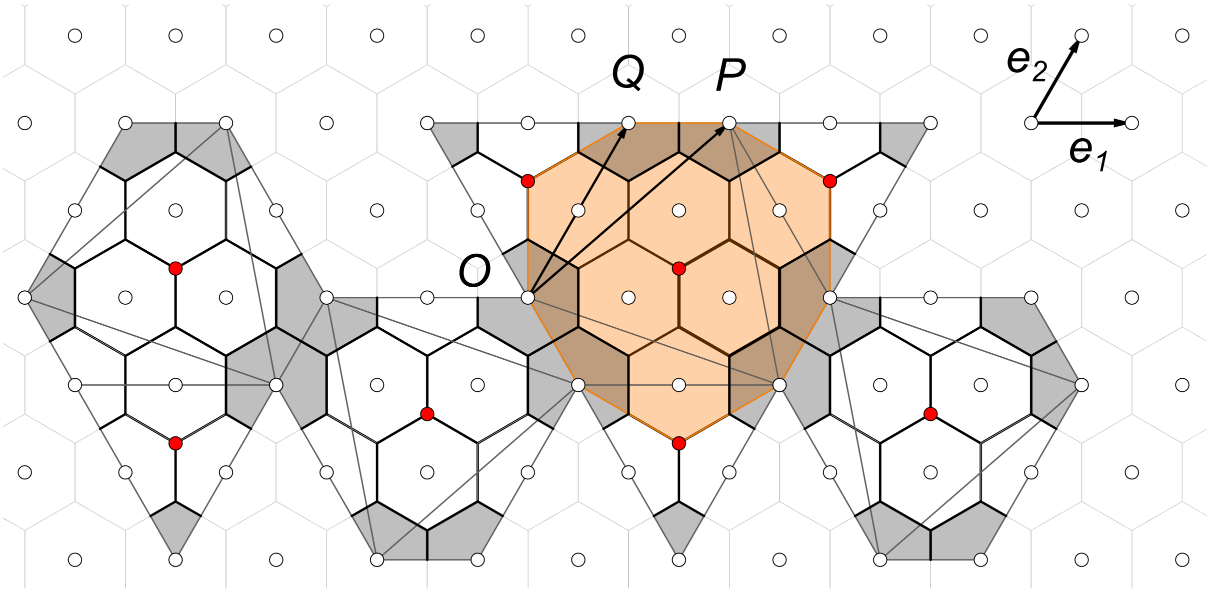

Figure S1: the full planar net of the C_{68} fullerene graph with T_d symmetry, corresponding to the nanoparticle with $N = 180$ in Fig. S2.

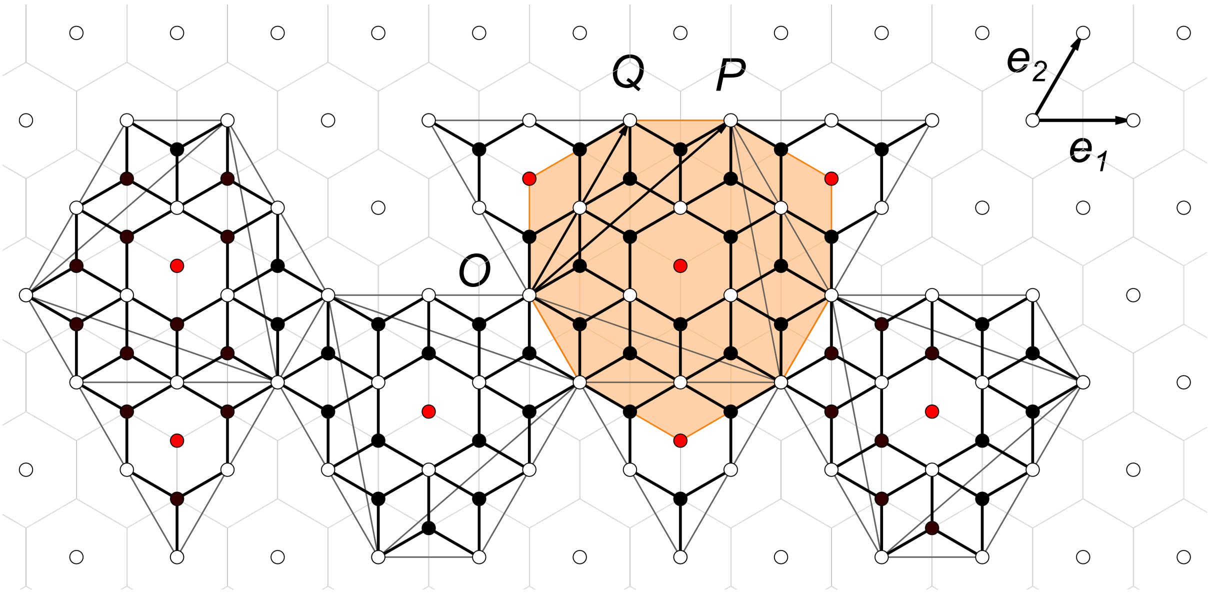

Figure S2: the full planar net of the nanoparticle graph with T_d symmetry, corresponding to the fullerene graph C_{68} in Fig. S1.

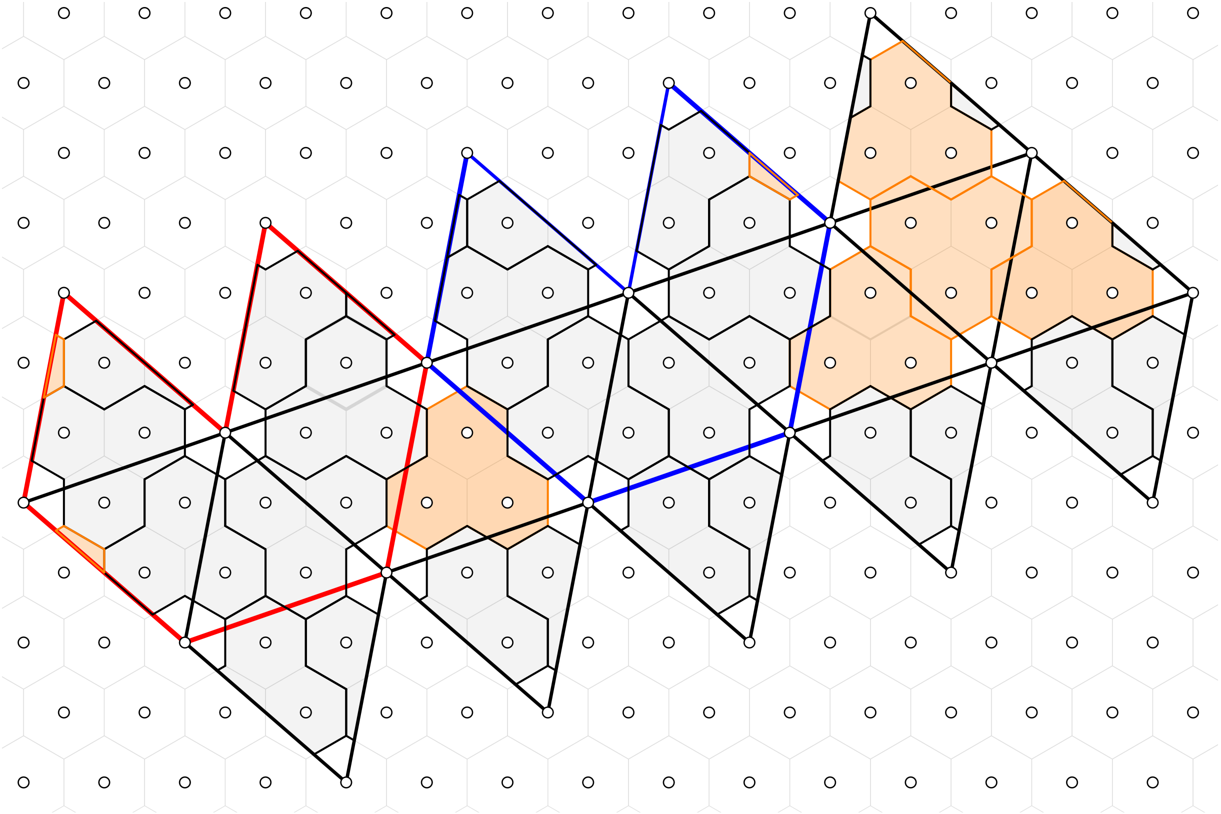

Figure S3: The figure demonstrates all options in which the symmetry of an icosahedral particle C_{140}, corresponding to a nanoparticle with 360 chains, can be broken. By the vertex coloring rule the fullerene graph can be decorated by coloring exactly one vertex for each hexagonal face and none for the pentagonal faces, which corresponds to tiling the fullerene graph by triplets of hexagons sharing a vertex (i.e., the colored vertex). With regard to the icosahedral net for C_{140}, these tiles composed by triplets can only lie in two positions, either overlapping four triangular faces of the icosahedron (orange triplets), or overlapping three triangular faces of the icosahedron (grey triplets). A tessellation given by all orange triplets would maintain icosahedral symmetry in the decorated particle.
